# Supraspinal characterization of the thermal grill illusion with fMRI

**DOI:** 10.1186/1744-8069-10-18

**Published:** 2014-03-11

**Authors:** Albert Leung, Shivshil Shukla, Eric Li, Jeng-Ren Duann, Tony Yaksh

**Affiliations:** 1 Department of Anesthesiology, University of California, San Diego, School of Medicine, 9500 Gilman Drive, MC 0818, 92093 La Jolla, CA, USA; 2 Veterans Administration San Diego Healthcare System, 9300 Campus Point Drive, MC 7651, 92037 La Jolla, CA, USA; 3 University of California, San Diego, School of Medicine, 92037 La Jolla, CA, USA; 4 Swartz Center for Computational Neuroscience, Institute for Neural Computation, University of California, San Diego, 9500 Gilman Drive # 0559, 92093-0559 La Jolla, CA, USA

**Keywords:** Thermal grill illusion, Neuropathic pain, Sensory integration, Spatial summation, fMRI

## Abstract

**Background:**

Simultaneous presentation of non-noxious warm (40°C) and cold (20°C) stimuli in an interlacing fashion results in a transient hot burning noxious sensation (matched at 46°C) known as the thermal grill (TG) illusion. Functional magnetic resonance imaging and psychophysical assessments were utilized to compare the supraspinal events related to the spatial summation effect of three TG presentations: 20°C/20°C (G2020), 20°C/40°C (G2040) and 40°C/40°C (G4040) with corresponding matched thermode stimuli: 20°C (P20), 46°C (P46) and 40°C (P40) and hot pain (HP) stimuli.

**Results:**

For G2040, the hot burning sensation was only noted during the initial off-line assessment. In comparison to P40, G4040 resulted in an equally enhanced response from all supraspinal regions associated with both pain sensory/discriminatory and noxious modulatory response. In comparison to P20, G2020 presentation resulted in a much earlier diminished/sedative response leading to a statistically significantly (P < 0.01) higher degree of deactivation in modulatory supraspinal areas activated by G4040. Granger Causality Analysis showed that while thalamic activation in HP may cast activation inference in all hot pain related somatosensory, affective and modulatory areas, similar activation in G2040 and G2020 resulted in deactivation inference in the corresponding areas.

**Conclusions:**

In short, the transient TG sensation is caused by a dissociated state derived from non-noxious warm and cold spatial summation interaction. The observed central dissociated state may share some parallels in certain chronic neuropathic pain states.

## Background

Simultaneous presentation of non-noxious warm (40°C) and cold (20°C) stimuli in an interlacing fashion results in a transient paradoxical noxious sensation known as the thermal grill (TG) illusion for the observer [1]. Despite its well known illusive characteristics, the mechanisms underlying this paradoxical phenomenon still remain elusive [2,3]. Some believe that the TG phenomenon shares certain parallels in central pain states [3,4]. Early TG studies have shown supraspinal involvement including the thalamus (TH) and anterior cingulate cortex (ACC), suggesting a disruption of thermosensory integration [2,5]. However, some key questions regarding this process of sensory disruption remain unanswered. One of these questions is how the spatial summation effect of the TG in either single or mixed temperatures will affect supraspinal regions encoding acute noxious or non-noxious thermal sensations. Other mechanisms related questions include how the mixed temperature (20°C/40°C) TG presentation will influence the causality relationship in the supraspinal acute pain network. Based on previous studies, the supraspinal acute thermal pain network consistently involves seven regions: primary and secondary somatosensory (SSC1. SSC2), insular cortex (IN), ACC, prefrontal cortices (PFCs) and TH [6]. Among those regions, SSC1 and SSC2 are commonly linked to the sensory/discriminatory aspect of pain processing, whereas, ACC is associated with the affective pain experience [7]. In addition, various regions of PFCs are related to attention and pain modulatory functions, and the IN is implicated in assessing the magnitude of pain [6,8,9]. Furthermore, the inferior parietal lobe (IPL) is also known to play an important role in spatial discriminatory functions of thermal pain perception [10-12]. From the afferent input standpoint, A-delta fibers mediated afferent modalities such as cold or punctate (acupuncture) stimuli can have a profound deactivational effect on the supraspinal regions encoding or modulating pain [13,14]. It is likely that this non-noxious cold mediated deactivational effect can be enhanced by the TG spatially summative presentation, thus interfering the supraspinal modulatory function normally associated with warm thermosensory perception. In a previous study, the transient nature of the grill sensation was qualitatively characterized as predominantly “hot” and “burning” and quantitatively matched the thermal sensation delivered via a thermode at around 46°C [15], suggesting the illusive sensation may derive from an augmented sensory perception to a spatially summated non-noxious warm stimulus. Thus , the authors hypothesized that functionally, a dissociated state consisting of decreased activities in PFCs and increased activities in supraspinal sensory/discriminatory regions would be observed with the TG mixed temperature presentation in comparison to the matched temperature.

With these understandings in the supraspinal acute hot pain (HP) network and early behavioral characterization of the TG, the authors hypothesized the following:

1) Spatial summation effect of the TG at 20°C (G2020) will result in less activation or more deactivation in supraspinal areas (PFCs) related to HP modulation in comparison to thermode cold stimulus at 20°C (P20);

2) Spatial summation effect of TG at 40°C (G4040) will result in more supraspinal activation in the sensory/discriminatory aspect of acute HP processing in comparison to the matched thermode stimulus at 40°C (P40);

3) Mixed TG summation effect at 20°C and 40°C (G2040) will result in less activation (more deactivation) in areas of PFCs(modulatory) and more activation in areas of sensory/discriminatory or affective response in comparison to matched thermode stimulus at 46°C (P46).

Correlating with psychophysical assessment, this study utilized functional magnetic resonance imaging (fMRI) to test the stated hypotheses by comparing the grill stimuli with matching stimuli from the thermal probe, and assessing the functional connectivity and causality of acute thermal pain related supraspinal network in the three different TG presentations.

## Results

Eighteen subjects underwent the initial off-line assessment. Fifteen right-handed dominant subjects (7 males) with the age range from 20 to 55 years old who reported hot burning sensation during the initial off-line assessment were subsequently enrolled for the fMRI study. Two subjects (one male and one female) did not tolerate the study duration in the scanner. As a result, only data from the thirteen subjects who completed the entire study was used for analysis.

### Psychophysical assessment

#### Qualitative description

All subjects who underwent the fMRI study felt hot burning sensation with G2040 presentation in the initial off-line assessment. However, in the final (on-line) assessment, no hot burning pain was reported in the G2040 presentation with twelve of the thirteen subjects felt a combination of warm and cold sensations, and one subject felt predominantly warm sensation. In addition, only warm and cold sensations were reported in G4040 and G2020 and the matched thermode paradigms respectively in the off-line (initial and final) assessments. In the post-scanning online assessment, all subjects reported a transient hot burning sensation associated with the G2040 presentation. No pain or burning sensation was reported with G2020 and G4040 and their matched temperature thermode paradigms.

#### Thermal threshold and noxious sensation rating

The average pre-scanning thresholds (°C ± SD) for cold, warm, cold pain and hot pain for the subjects (n = 13) were 28.1 ± 1.8, 36.3 ± 2.1, 11.2 ± 10.3 and 48.6 ± 1.3 respectively. The pre-scanning (off-line) average initial VAS scores (±SD) for HP (48.1 ± 4.2) were significantly (P < 0.01) higher than the pain VAS scores of P46 (42.7 ± 3.9) and G2040 (42.5 ± 3.5). The pre-scanning final VAS score of HP remained significantly (P < 0.01) higher than P46 at the final offline assessment, and no noxious feeling was reported with G2040. No noxious sensation was reported with P20, P40 G2020 and G4040 paradigms in the pre-scanning assessments. In the post-scanning (on-line) assessment overall noxious sensations were reported in the HP, P46 and G2040 with NPRS scores at 6.75 ± 0.52, 3.50 ± 0.41, and 1.95 ± 0.52 respectively.

### fMRI within-group random effect

#### Thermode HP

**Figure 1:**
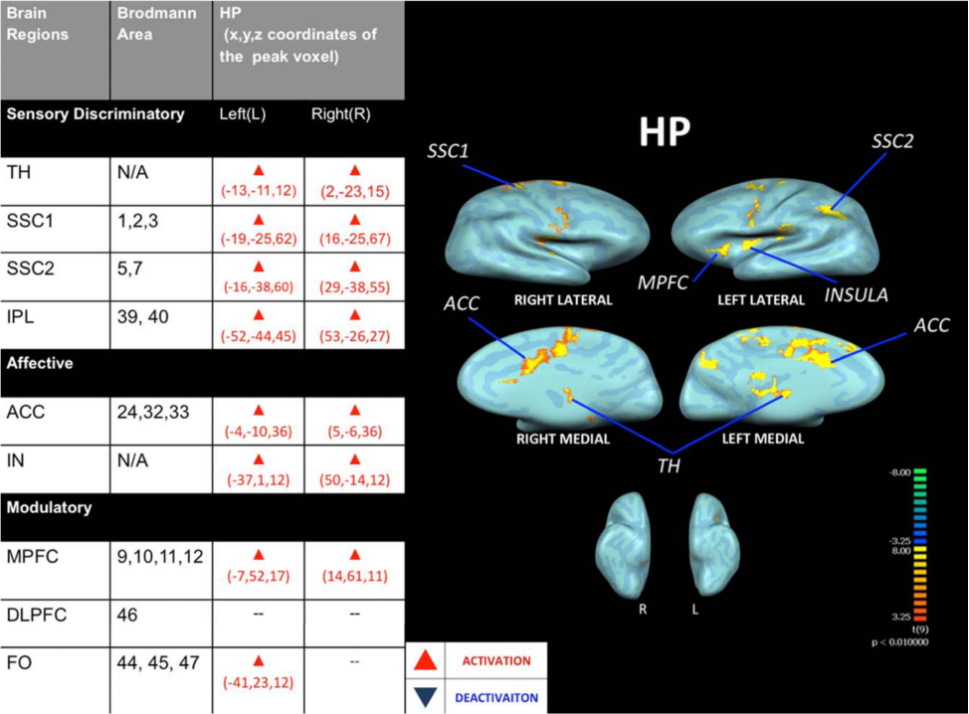
**Supraspinal activities (P < 0.01,cluster threshold > 150) of heat pain (HP) delivered via thermode.** TH: Thalamus; SSC1: Primary Somatosensory Cortex; SSC2: Secondary Somatosensory Cortex; IPC: Inferior Parietal Lobe; ACC: Anterior Cingulate Cortex; IN: Insular Cortex; DLPFC: Dorsolateral Prefrontal Cortex; MPFC: Medial Prefrontal Cortex; FO: Frontal Operculum.

Baseline within-group HP paradigm random effect analysis demonstrated significant (P < 0.01, cluster threshold > 150) activations in supraspinal areas (SSC1, SSC2, TH, ACC, IN and PFCs) related to acute thermal pain sensory discriminatory processing, affective and neuromodulatory response (see Figure 1).

#### Thermal grill (G2020, G4040, G2040)

**Figure 2:**
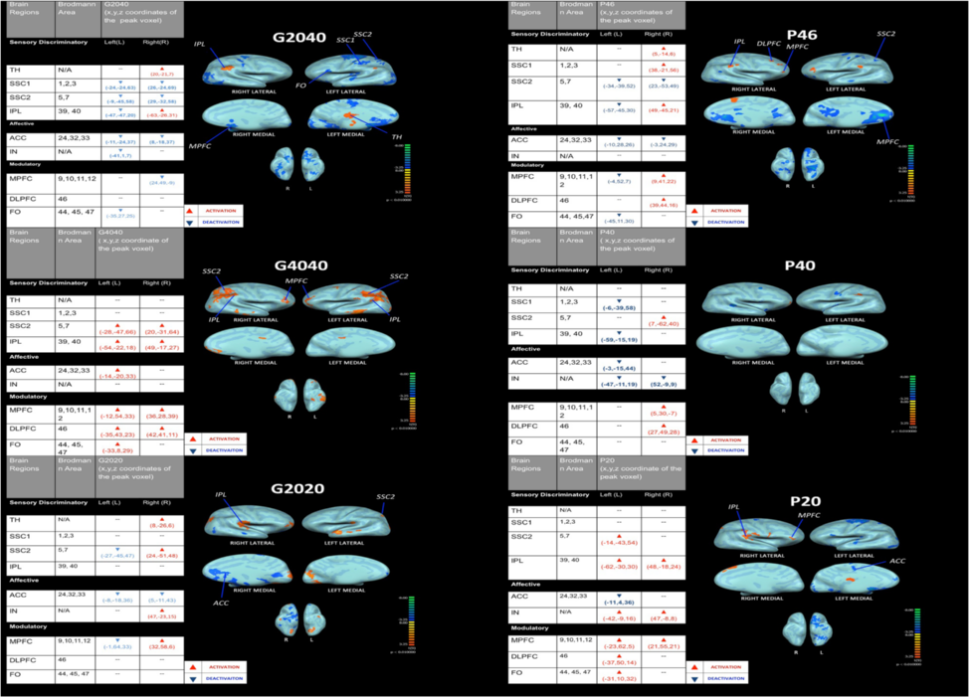
**Supraspinal activities (P < 0.01,cluster threshold > 150) of thermal grill (G2020, G2040, G4040) and thermode (P20, P46, P40).** TH: Thalamus; SSC1: Primary Somatosensory Cortex; SSC2: Secondary Somatosensory Cortex; IPC: Inferior Parietal Lobe; ACC: Anterior Cingulate Cortex; IN: Insular Cortex; DLPFC: Dorsolateral Prefrontal Cortex; MPFC: Medial Prefrontal Cortex; FO: Frontal Operculum.

Within-group random effect analysis of G2020 illustrated a significant (P < 0.01, cluster threshold > 150) degree of deactivation pattern in left SSC2, bilateral ACC and left MPFC; and activation in right TH, SSC2 and IN(see Figure 2 and Table 1).

**Table 1:** Summary table for paradigms: Thalamus; SSC1: primary somatosensory cortex; SSC2: secondary somatosensory cortex; IPC: inferior parietal lobe; ACC: anterior cingulate cortex; IN: insular cortex; DLPFC: dorsolateral prefrontal cortex; MPFC: medial prefrontal cortex; FO: frontal operculum; L: left; R: right; +: activation; −-: deactivation; /: no activation or deactivation

	**HP**		**G2040**		**G4040**		**G2020**		**P46**		**P40**		**P20**	
	**L**	**R**	**L**	**R**	**L**	**R**	**L**	**R**	**L**	**R**	**L**	**R**	**L**	**R**
**TH**	+	+	/	+	/	/	/	+	/	+	/	/	/	/
**SSC1**	+	+	--	--	/	/	/	/	/	+	--	/	/	/
**SSC2**	+	+	--	--	+	+	--	+	--	--	/	+	+	/
**IPL**	+	+	--	+	+	+	/	/	--	+	--	/	+	+
**ACC**	+	+	--	--	+	/	--	--	--	--	--	/	--	/
**IN**	+	+	--	/	/	/	/	+	/	/	--	--	+	+
**MPFC**	+	+	/	--	+	+	--	+	--	+	/	+	+	+
**DLPFC**	/	/	/	/	+	+	/	/	/	+	/	+	+	/
**FO**	--	--	--	/	+	/	/	/	--	/	/	/	+	+

Within-group random effect analysis of G4040 revealed an overall activation pattern similar to the HP paradigm in all areas associated with acute thermal processing. In contrast with G4040, within-group random effect analysis of G2040 demonstrated an overall deactivation pattern in supraspinal regions associated with acute thermal pain processing except for the right inferior parietal lobe and bilateral TH in which an activation (P < 0.01) pattern was noted. PFCs such as MPF were deactivated in G2040 (see Figure 2 and Table 1).

#### Thermal probe (P20, P40, P46)

Within-group random effect analysis of P20 stimulation demonstrated significant (P < 0.01, cluster threshold > 150) activations in bilateral parietal areas and ipsilateral (left) SSC2. In addition, significant activations in bilateral MPFC, left frontal operculum and DLPFC were noted. In the affective areas, activations in the bilateral IN were noted, whereas ACC was deactivated (see Figure 2 and Table 1).

Within-group random effect analysis of the P40 paradigm resulted in significant (P < 0.01, cluster threshold > 150) activation of the right SSC2. In the modulatory areas, the right DLPFC was activated, whereas, in the brain areas (ACC, IN) encoding for affective aspect of pain perception was primarily deactivated (see Figure 2 and Table 1).

Within-group random effect analysis of P46, the thermode temperature matched the thermal grill sensation at G2040, demonstrated significant (P < 0.01, cluster threshold > 150) activations in the contralateral(right) somatosensory areas (TH, SSC1 and inferior parietal areas), and modulatory areas including frontal operculum and MPFC. No overall activations were observed in the affective supraspinal components (see Figure 2 and Table 1).

### Between-group random effect analysis

#### G2020 > P20 (hypothesis#1)

In the between-group random effect analysis (G2020 > P20, see Table 2), G2020 stimulation resulted in significantly (P < 0.01, cluster threshold > 150) less activities in several pain modulatory areas including the MPFC and frontal operculum, as well as sensory/discriminatory areas in inferior parietal lobe (Table 2).

**Table 2:** Significant (P < 0.01, cluster threshold > 150) findings in G2020 > P20 between-group random effect analysis

**Regions of activities**	** Peak T-value**	**Cluster size**	**Brodmann area**	**Peak voxel coordinates**		
**Right hemisphere**				**X**	**Y**	**Z**
MPFC	−3.42127	843	11	32	31	−3
IPL	−2.797605	425	39	44	−44	30
**Left hemisphere**				**X**	**Y**	**Z**
DLPFC	−3.557824	547	9	−31	40	27
FO	−3.528314	332	10	−37	50	−6

*IPL* Inferior Parietal Lobe, *DLPFC* Dorsolateral Prefrontal Cortex, *MPFC* Medial Prefrontal Cortex, *FO* Frontal Operculum.

#### G4040 > P40 (hypothesis #2)

Between-group random effect analysis (G4040 > P40, see Table 3) demonstrated that G4040 resulted in significantly (P < 0.01, cluster threshold > 150) higher and larger areas of activities in supraspinal areas associated with sensory discriminatory (SSC2, IPL) aspects of pain perception in comparison to P40. However, these elevated activities in G4040 were matched with equally enhanced activities in the modulatory supraspinal areas (MPFC).

**Table 3:** Significant (P < 0.01, cluster size > 150) findings in G4040 > P40 between-group random effect analysis in HP related supraspinal regions

**Regions of activities**	** Peak T-value**	**Cluster size**	**Brodmann area**	**Peak coordinates**		
**Right hemisphere**				**X**	**Y**	**Z**
MPFC	3.065635	329	9	17	28	30
SSC2	3.571226	2367	5	14	−47	57
**Left hemisphere**				**X**	**Y**	**Z**
SSC2	3.153579	428	7	−19	−50	60
SSC2 & IPL	3.338459	5408	7, 39, 40	−37	−41	36

*TH* Thalamus, *SSC1* Primary Somatosensory Cortex, *SSC2* Secondary Somatosensory Cortex, *IPC* Inferior Parietal Lobe, *ACC* Anterior Cingulate Cortex, *IN* Insular Cortex, *DLPFC* Dorsolateral Prefrontal Cortex, *MPFC* Medial Prefrontal Cortex, *FO* Frontal Operculum.

#### G2040 > P46 (hypothesis #3)

Between-group random effect analysis (G2040 > P46, see Table 4) demonstrated that G2040 presentation resulted in significantly (P < 0.01, cluster threshold > 150) higher activities than P46 in several supraspinal areas (IPL and ACC) related to sensory discriminatory and affective aspects of HP perception. However, in comparison to P46, G2040 resulted in significantly (P < 0.01, cluster threshold > 150) less activities in the pain related supraspinal modulatory areas (MPFC).

**Table 4:** Significant (P < 0.01, cluster threshold >150) for G2040 > P46 between-group random effect analysis in HP related supraspinal regions

**Regions of activities**	** Peak T-value**	**Cluster size**	**Brodmann area**	**Peak coordinates**		
**Right hemisphere**				**X**	**Y**	**Z**
IPL	3.210518	298	39	48	−53	24
**Left hemisphere**				**X**	**Y**	**Z**
MPFC	−4.075896	209	9	−18	47	32
ACC	3.545434	277	32	−5	41	13

*IPL* Inferior Parietal Lobe, *ACC* Anterior Cingulate Cortex, *MPFC* Medial Prefrontal Cortex.

#### HP > P46 and HP > P40

As shown in Table 5, HP generally resulted in a significantly (P < 0.01, cluster threshold > 150) higher activation in supraspinal regions associated with acute pain perception in comparison to either P46 or P40 with the exception of right MPFC. In comparison to the HP contrast, less significant (P < 0.05, cluster threshold > 150) activation difference (P46 > P40) was observed in the right IN, ACC and left SSC1.

**Table 5:** Between-group comparison of thermode stimuli including heat pain (HP), 46°C (P46) and 40°C (P40)

**Regions of activities related to HP (peak voxel x,y,z coordinates)**	**Within-group random effect analysis peak voxel T-value**			**Between-group (cluster threshold > 150) comparison P-value**		
	**HP**	**P46**	**P40**	**HP > P40**	**HP > P46**	**P46 > P40**
**Left hemisphere**						
**SSC I (−19,-25,62)**	17	−3.66	−4.42	★★	★★	★
**SSC 2 (−16,-38,60)**	19.76	−2.93	−3.68	★★	★★	
**IN (−37,1,12)**	22.6	−2.3	−2.9	★★	★★	
**TH (−13,-11,12)**	16.6	2.08	−4.78	★★	★★	
**Right hemisphere**						
**SSC I (16,-25,67)**	38.64	2.19	3	★★	★★	
**ACC (5,-6,36)**	13.02	−2.3	−2.82	★★	★★	★
**IN (50,-14,12)**	16.74	2	−3.5	★★	★★	★
**MPFC (14,61,11)**	5.76	3.94	0.92			
**TH (2,-23,15)**	8.19	3.43	1.37	★★	★★	

*SSC1* Primary Somatosensory cortex, *SSC2* Secondary Somatosensory Cortex, *ACC* Anterior Cingulate Cortex, *IPL* Inferior Parietal Lobe, *ACC* Anterior Cingulate Cortex, *MPFC* Medial Prefrontal Cortex, *IN* Insular Cortex, *TH* Thalamus. ★: P < 0.05, ★★: P < 0.01.

### Granger causality analysis

In exploring the causality relationship among HP related supraspinal regions, GCA was first conducted in the HP and compared with G2040:

#### HP and G2040

**Figure 3:**
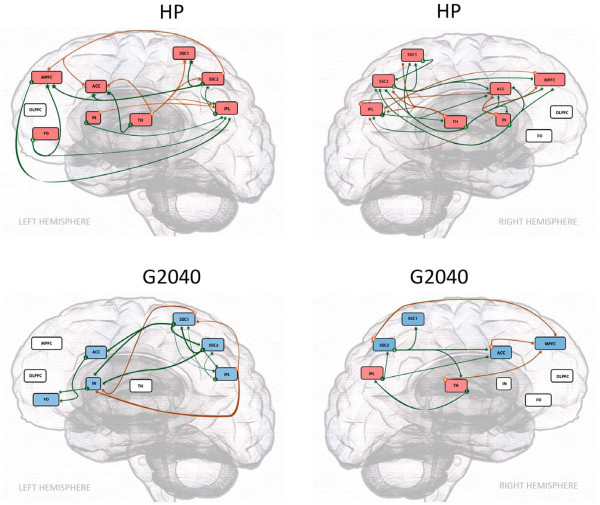
**Granger causality analysis of heat Pain (HP) and thermal grill 20°C/40°C (G2040).** Red boxes -indicate brain regions with significant (P < 0.01) activation, whereas blue boxes indicate brain regions with significant (P < 0.01) deactivation. Green lines (L) indicate the inference occurs towards the left hemisphere from regions of the shown hemisphere, whereas, orange lines (R) indicate the inference occurs towards the right hemisphere from regions of the shown hemisphere. TH: Thalamus; SSC1: Primary Somatosensory Cortex; SSC2: Secondary Somatosensory Cortex; IPC: Inferior Parietal Lobe; ACC: Anterior Cingulate Cortex; IN: Insular Cortex; DLPFC: Dorsolateral Prefrontal Cortex; MPFC: Medial Prefrontal Cortex; FO: Frontal Operculum.

Although both paradigms consisted of activation in the TH, the causality inference from the TH to other pain related supraspinal regions was noticeably different between the two paradigms (see Figure 3). While TH activation in both paradigms casted direct inference on the IPL, only HP stimulus resulted in activation inference in all other pain related somatosensory, affective and modulatory areas, whereas G2040 thalamic activation resulted in deactivation inference in the corresponding areas. In the HP paradigm, it was noted that the activation of the TH led to the activation of SSC2 and ACC, whereas the activation of TH in the G2040 paradigm led to the deactivation of the ACC and SSC2. Furthermore, in HP, the activation of modulatory areas such as MPFC and other PFCs casted a direct influence in the IPL, whereas in G2040, these corresponding modulatory supraspinal areas were mostly in deactivated states and had no direct influence on the IPL.

Further GCA was conducted to assess the causality relationship in G2020 and G4040 with comparison to G2040:

#### GCA of G2020, G2040 and G4040

**Figure 4:**
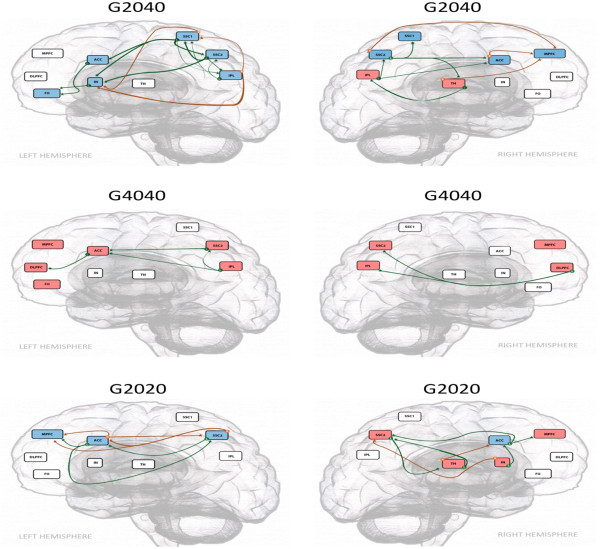
**Granger causality analysis of three different thermal grill presentations (G2020, G2040, G4040).** Red boxes -indicate brain regions with significant (P < 0.01) activation, whereas blue boxes indicate brain regions with significant (P < 0.01) deactivation. Green lines (L) indicate the inference occurs towards the left hemisphere from regions of the shown hemisphere, whereas, orange lines (R) indicate the inference occurs towards the right hemisphere from regions of the shown hemisphere. TH: Thalamus; SSC1: Primary Somatosensory Cortex; SSC2: Secondary Somatosensory Cortex; IPC: Inferior Parietal Lobe; ACC: Anterior Cingulate Cortex; IN: Insular Cortex; DLPFC: Dorsolateral Prefrontal Cortex; MPFC: Medial Prefrontal Cortex; FO: Frontal Operculum.

Inference analysis was conducted in the supraspinal regions related to HP perception in all three TG paradigms. In G2020, unilateral right thalamic activation was observed and casted a direct inference on the deactivation of right ACC and left SSC2 which further imposed a direct inference on the deactivation of bilateral ACC. No significant activities of IPL were detected for any further inference analysis. No significant inference detected from PFCs as well. In G4040, activation of bilateral IPL and SSC2 appeared to be independent of the TH activities (no significant activation was detected) and casted a direct activation inference on the right ACC, which in turn activated various PFCs. The activated PFCs (FO and DLPFC) then casted direct inferences on SSC2 and IPL. In contrast to G4040, thalamic activation in G2040 led to left ACC and right MPFC deactivation. This right thalamic activation, also observed in G2020 but not in G4040, led to the activation of left IPL. However, unlike G4040, no inference of PFCs on the IPL was detected in G2040. Although the left frontal operculum activation in G2040 led to the deactivation of left ACC, no direct inference of other PFCs was observed on the activated inferior parietal regions (see Figure 4).

The authors asserted that the above observed inference was not simply due to a difference in the hemodynamic response as the pattern of inference was remarkably different with various stimulation paradigms. To further confirm these functional relationship in the supraspinal regions associated with various thermal grill paradigms, we further tested their connectivity with Psychophysiological Interactions (PPI) Analysis. Since the right IPL was implicated in thermal pain perception and was found activated in both G4040 and G2040 paradigms, it was adopted as the seeded region in the PPI analysis in both grill paradigms. In G2040, the right IPL demonstrated a strong functional connectivity to regions including ACC, and MPFC and SSC2 as observed in GCA. However, in G4040, the right IPL did not demonstrate a significant level of functional connectivity to these regions, suggesting an important role that the right IPL play in thermal grill illusion. These additional functional connectivity findings support the result of GCA in which the right IPL consisted of a direct inference on ACC and SSC2.

## Discussion

The findings of the current study indicate that the transient sensation of the TG illusion derived from a unique a pattern of supraspinal events associated with the spatial summation effect of the grill. Although the simultaneous presentation of non-noxious cold and warm stimuli in close proximity resulted in deactivational response in supraspinal regions (except IPL) normally associated of somatosensory discrimatory function, the presentation also resulted in significant deactivation in regions including MPFC, DLPFC and FO which are normally associated for pain modulation. This degree of deactivation was not observed in other two grill paradigms and matched thermal probe paradigms. The current findings therefore suggest that this generalized deactivation in supraspinal pain modulation accompanied by partial somatosensory activation as observed in G2040 resulted in the paradoxical heat pain illusion. The current study therefore provides novel information on the mechanisms of the thermal grill illusion and the effect of spatial summation on thermal perception.

Recent studies with peripheral sensory testing and functional imaging techniques have provided insightful information regarding areas of the central nervous systems involved in encoding acute and chronic pain [16-20]. These supraspinal regions include the SSC1 and SSC2, TH, IN, amygdala, and PFCs [21-27]. In addition other supraspinal regions such as the PFCs are known to play a crucial role in pain modulation [28-31]. In the area of acute thermal pain perception, several supraspinal regions (TH, SSC1, SSC2, IPL, ACC, IN , PFCs) were consistently being implicated [6]. While SSC1 and SSC2 are commonly linked to the sensory/discriminatory aspect of pain processing, ACC is associated with the affective pain experience [7]. While various regions of PFCs such as MPFC, DLPFC and FO are related to attention and pain modulatory functions, the IN is implicated in assessing the magnitude of pain and modulation as well [6,8,9]. The anterior IN is more strongly functionally connected to areas known for affective and cognitive processing but the posterior IN is more strongly connected with areas known for sensory-discriminative processing of noxious and somatosensory stimuli [32]. Furthermore, the IPL is also known to play an important role in spatial discriminatory functions of thermal pain perception [10-12]. While BOLD signals in bilateral IPLs were specifically correlated with the ratings of innoxious heat stimuli, responses in the SSC1 and SSC2 were correlated with pain intensity [33]. Despite this understanding in the supraspinal pain signal decoding process in healthy subjects, specific functional connectivity alteration does occur in neuropathic pain states [34,35]. This current understanding in the supraspinal mechanisms leading to the acute thermal pain perception and modulation provides a framework for studying sensory integration such as the TG illusion [36]. Several Important observations derived from the current study that may further the understanding in the underlying mechanisms of pain perception and TG illusion.

### Stimulation intensity and supraspinal modulatory response

First, noxious HP stimulation resulted in activation in supraspinal regions corresponding to sensory/discriminatory and affective functions as observed in HP paradigm (see Figure 1). The observed areas of activities were similar to published studies [6]. In comparison to P46 and P40, the degree of sensory discriminatory decoding/response was significantly more robust with HP (48 ± 1.6°C) as demonstrated in the between-group comparison (see Table 4). However, in the same comparison, the modulatory response associated with HP was not more significantly elevated than P46 and P40. Although a trend of enhancing response from the MPFC to the rising thermal intensities ranging from P40 to HP (Tables 4 &5) was observed, it was not significantly different among the three paradigms (P40, P46 and HP). In the causality analysis of HP, various pain modulatory regions such as the PFCs may cast direct inference on the supraspinal sensory/discriminatory regions (SSC2 and IPL) with IN casting direct inference to ACC, MPFC and all sensory discriminatory regions, whereas, affective regions (ACC) may cast no direct inference on those regions (see Figure 3), suggesting that an indirect feedback loop involving an affective region mediated pain modulatory effect on sensory/discriminatory aspect of pain processing. This combined result suggests that pain perception may stem from a disproportional response found between sensory/affective and modulatory decoding of the stimulus. In other words, there may be an intrinsic limitation in the supraspinal modulatory response to the intensity of the stimulation. If the intensity of the stimulation exceeds the intrinsic ability of modulation, pain perception occurs.

### The spatial summation effect of TG for warm and cold

In assessing the spatial summation effect of the TG which provided a much larger area of skin surface contact in comparison to the thermode, warm sensory summation from G4040 resulted in a significantly enhanced response in supraspinal sensory discriminatory regions, especially in SSC2 and IPL in comparison to P40 as shown in the between-group comparison (see Table 3). While P40 only induced activation in the right IPL, G4040 caused bilateral SSC2 and IPL activation. This enhanced sensory discriminatory response from G4040 was proportionally counter-reacted with augmented supraspinal modulatory response from the PFCs, which in the causality analysis was found to impose an inference on the SSC2 and IPL (see Figure 4). These closely correlated interactions between modulatory and sensory discriminatory components of pain resulted in unremarkable affective response, and thus no noxious feelings were reported by the observers in G4040. This observation is in line with previous studies in which warm/heat related spatial summation causes significant temporal and spatial shift in supraspinal regions related to thermal pain processing [37] and the probability of obtaining SSC2 activation appears related to the total amount of body surface stimulated (spatial summation) [38].

On the other hand, spatially summated cold stimulus (G2020) resulted in a statistically significant diminished response in all three components of pain perception in comparison to P20 presentation (see Table 1). In the GCA of G2020 (see Figure 4), unilateral right thalamic activation may lead to the deactivation of both the right ACC and the left SSC2, which in itself further imposed a direct inference on the deactivation of bilateral ACC. However, in the case of G2020, neither significant activity of IPL nor significant inference from PFCs on other HP related supraspinal regions were detected. This observed deactivational effect of the cold summation in G2020 appeared to have a direct effect on the thermal sensory perception when both thermal (cold and warm) modalities were presented simultaneously. While thalamic activation in HP may cast activation inference in all hot pain related somatosensory, affective and modulatory areas, similar activation in G2040 and G2020 may result in deactivation inference in the corresponding areas. Lindstedt et al.’s study has postulated the role of thalamus in the illusion. The findings from the current study further confirms the sensory integration role of the TH in the lateral and medial supraspinal systems and the process of generating the TG sensation as postulated in the previous study [5,26]. Like G4040, IPL activation was observed in G2040. However unlike G4040, supraspinal areas normally associated with pain modulatory functions became significantly deactivated or less activated in G2040 and may cast no modulatory inference and functional connectivity on the IPL as assessed with GCA and PPI analysis, suggesting an imbalance between modulatory and sensory/discriminatory functions in G2040.

#### Effect of grill mixed temperature on supraspinal response

While the activation of modulatory areas such as MPFC in the HP paradigm may cast a direct influence on the parietal areas as demonstrated with the causality analysis, the corresponding modulatory components were mostly in deactivated states and had no direct influence on the parietal areas in the G2040 presentation (see Figure 4). This unique deactivation pattern was not observed in P46, the matched temperature for the grill sensation, but similarly present in the G2020 (see Figure 4). This observed contrast between G2040 and P46 further supports the notion that a dissociated state between modulatory and somatosensory supraspinal functions is present in the illusion. Therefore cold summation(G2020) leads to a generalized diminished modulatory response from MPFC, DLPFC and FO which are otherwise enhanced in warm summation(G4040) with the presence of enhanced activities in SSC2 and IPL while warm summation Consequentially, a short transient enhanced perception of warm summation occurs as a hot burning sensation known as the TG illusion.

#### Study related issues

Several study related issues are worthy of discussion. First, in the current study, psychophysical assessment was intentionally not performed simultaneously with functional imaging data acquisition as this approach assessment was deemed infeasible for the transient nature of TG sensation. Instead, pre- and post- scanning assessments (as utilized in a previous study [14]) were conducted to minimize the potential interference on the functional imaging data, and thus increasing the specificity of the functional data interpretation. The sample size, although small, is comparable to a previous published TG study and most published functional imaging studies related to thermal pain [2,38]. The relative high homogeneity in psychophysical response in regards to the grill sensation further minimized the concern for the relative small sample size. Functional connectivity findings with PPI analysis also supported the causality inference findings with GCA and overall interpretation of the result.

## Conclusion

In short, the transient hot burning sensation associated with the TG presentation likely derives from a dissociated state between the DLPFC, MPFC and FO (modulatory regions) and IPL (sensory discriminatory regions) of thermal perception. This transient dissociated state is most likely caused by the interaction between warm and cold spatial summation.

## Methods

With the Institution Human Subject Protection Committee approval, healthy volunteers were screened and enrolled after informed consent was obtained from each subject for the study based on the following inclusion criteria: age range from 18 to 80; male and female; no analgesics taken for 2 weeks prior to the study; and absence of any acute or chronic pain states. These healthy subjects were recruited via advertisement (newspaper and flyers) The exclusion criteria included: history of psychological illness or claustrophobia; lack of ability to understand the experimental protocol or to adequately communicate in English; pregnancy and pending litigation. Subjects with a history of head injury, trauma or surgery to lower extremities or low back, and any metallic implant in the body were also excluded.

### Pre-scanning thermal threshold and psychophysical assessments

To be consistent with the stimulation site in the study, the location for the thermal thresholds measurement and all stimulations was marked at the medial aspect of the left calf between the 6^th^ and 7^th^ marking of an elastic band which consisted of a total of 13 increments, extending from the medial malleolus to the medial tibial plateau. Non-noxious and noxious thermal thresholds including cold and warm, cold and hot pain thresholds were measured by using a Thermal Sensory Analyzer (Medoc Advanced Medical Systems, Minneapolis). This device consisted of a thermode measuring 46 × 29 mm. The temperature of the thermode could either rise or fall (at a rate of 1.2°C/sec for cold and warm sensations, and 3°C/sec for cold and hot pain), depending on the sensations that were being tested. The subject signaled the onset of feeling the tested sensation by pressing a switch, which in turn reversed the temperature and returned the temperature of the thermode to the 32°C baseline. For the hot pain(HP) thresholds, the subjects pressed the switch when the rising temperature became noxious for them. The computer then recorded the temperature of the thermode when the switch was pressed. The average value of the testing result (4 trials for cold and warm, and 3 trials for cold and hot pain) was automatically calculated by the computer and displayed on the screen. This method of peripheral sensory testing has been well established in literature and has been used extensively in pain-related studies [15,39-42]. The determined subject specific hot pain threshold temperature was then used as the HP stimulus temperature during the fMRI study. While it might be ideal to solicit pain rating during the scanning for functional imaging correlation, the assessments could cause distraction to the subjects and thus negatively impacting the correlation of supraspinal response with the stimulation paradigms. Prior to conducting the study, the authors tested the pain assessment during scanning with continuous rating of pain and found that it was infeasible to assess the subjects continuously with both qualitative and quantitative measurement throughout the entire 30-second of stimulation. As determined in a previous study, the noxious aspect of TG sensation was most intense and noticeable in the initial 3–5 seconds of stimulation [15]. The authors adopted an alternative strategy described as “on-line and off-line assessments,” which was used in an earlier pain related functional imaging study [43] in which both pre- and post scanning assessments were conducted. First, independent pre-scanning (off-line) assessments were conducted for each subject prior to the scanning. The subjects were asked to first qualitatively characterize the sensation in the initial and last 6 seconds of the three TG presentations (presented in a random fashion with at least 10-minute washout period in between presentations) as warm, cold, neutral, hot, burning or non-burning at stimulation site in two separated 30-second stimulations for each presentation. Subjects reported hot burning sensation in the initial pre-scanning(off-line) assessments were then further assessed with fMRI scanning. Similar behavioral assessments were conducted for P20, P40, P46, and HP. The subjects were asked if pain was felt and if so, the intensity of pain was rated on a 0–100 Visual Analog Scale (VAS). In the post-scanning (on-line) assessment, the subjects were asked about the qualitative characterization of the stimuli and if the overall experience with the stimuli were painful. If so, they were asked to rate the intensity of pain on 0–10 Numerical Pain Rating Scale (NPRS) while the subjects were in the scanner [44].

### Thermal grill

**Figure 5:**
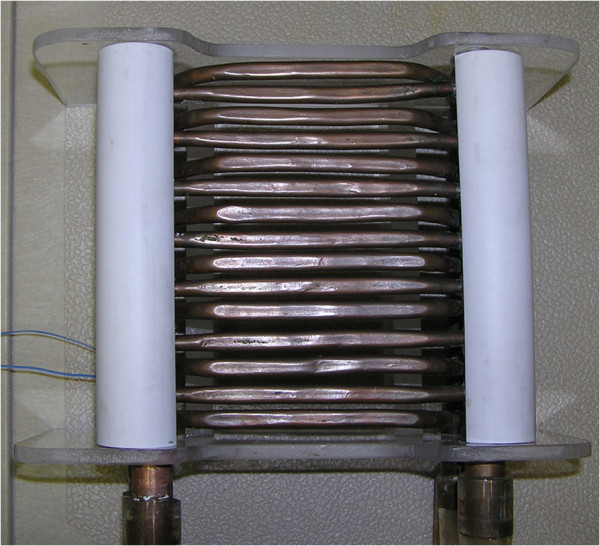
**The thermal grill consisted of two systems of 0.75 cm-flattened copper tubing cut into approximately 10 cm in length and placed one cm apart.** The inflow and outflow of the two separate systems were connected via a series of four-way valves to two separate heating and cooling water baths. The exact temperatures at the copper tubings were monitored with two separate temperature probes (Mallinckrodt Medical, Inc, St Louis, MO), which were attached to the copper tubings.

The thermal grill stimulator contained two systems of 0.75 cm flattened copper tubing cut approximately 10 cm in length and placed one cm apart. Each system consisted of five tubes. The systems were then mounted such that every other tubing was connected to a common inflow and outflow. The array was then mounted in a non-metallic frame of 15 cm × 20 cm (See Figure 5). The inflow and outflow of the two separate systems were connected via a series of four-way valves to two separate heating and cooling water baths. The exact temperatures at the copper tubing were monitored with two separate temperature probes (Mallinckrodt Medical, Inc, St Louis, MO), which were attached to the corresponding copper tubing. By appropriately adjusting the valves and settings of the temperatures in the water baths, the following running water temperature combinations (°C) were tested: 20/20, 40/40, and 20/40 [15]. The plastic material and copper tubings used for constructing the TG were non-ferromagnetic. The copper tubings were connected via one-inch plastic tubings to the water baths located outside the scanner. Prior to each scanning session, the connecting plastic tubings were primed with distilled water. The same system was used for a previous TG study in which the grill sensation was matched to 46°C [15]. During scanning, only the grill part of the system stayed inside the scanning room, whereas the water baths were placed outside the scanner room and connected to the grill via plastic tubings. This system was tested extensively in the MRI scanner prior to the current study to ensure that the TG setting created no magnetic interference on the acquired fMRI imaging from both a dummy sphere and two human volunteers.

### FMRI scanning

After the off-line assessment, subjects were placed comfortably in a supine position in a scanner with their eyes covered by an eye shield. A Facial-Cervical Collar Restraint Device was applied to minimize head movement [45]. The following 7 scanning paradigms were conducted in a random order for the TG (G2020, G4040, G2040) and thermode (HP, P20, P40, P46).

A block design stimulation paradigm consisting of 5 repetitions of a 60-second baseline/no stimulation followed by a 30-second thermal stimulation delivered to left calf area. In the HP paradigm, the stimulus was provided in an oscillating fashion at 1°C above or below the pre-determined subject-specific thresholds and the thermode temperature returned to 32°C during the baseline period. This method of HP stimulus was used in previous studies to minimize the concern of “wind-up” associated with prolonged painful stimulation and adaptation as subject’s rating of pain remain similar at each stimulation cycle [14]. In the TG (G2020, G4040, G2040) and matching thermode (P20, P40, P46) paradigms, 5 repetitions of 60 seconds of no stimulation and 30 seconds of stimulation were presented at the same marked location on the left calf. A post-scanning (on-line) pain assessment was conducted after each paradigm. In between scanning paradigms, a minimal of 15 minutes of washout period was provided to ensure any residual sensation from the preceding stimulation had completely subsided prior to the onset of the next stimulation paradigm. During the washout period, the subjects were asked to relax and reported to the investigators whether any residual sensations was present at a 3-minute increment while the investigators adjusted the thermode or TG temperature for the next paradigm. All subjects were informed about the duration of the fMRI study (about 2 hours)prior to the enrollment. They were provided the opportunities to empty their bladder prior to the scanning.

### Visual analogue scale

The Visual Analogue Scale (VAS) is a horizontal linear scale with the length of 100 mm. At one end of the scale is marked “No Pain” and at the other end of it is marked “Worst Pain Imaginable.” If the subject reports pain in testing, he or she would then be asked to mark the intensity of the painful sensation by drawing a perpendicular line across the linear scale. The length from the “no pain” end to the subject’s marking was then measured and recorded.

### fMRI parameters

FMRI Images were obtained via a 3 T GE scanner with T2*- weighted EPI-sequence (TE = 30 ms, TR = 2.0 s, α = 90°, Thickness = 4 mm, 32 slices, FOV = 256×256 mm^2^, MA = 64×64). A T1-weighted image (TR = 7.98 s, TE = 3.12 ms, Thickness = 1 mm, inter-slice distance = 1 mm, FOV = 256×256 mm^2^) was acquired for anatomical co-registration.

### Psychophysical data analysis

A 2-tailed T-test with Bonferroni correction was conducted in SPSS Statistics 17.0 platform to compare the VAS scores of noxious sensation detected in both thermode and grill sensations.

### fMRI data analysis

Each individual subject’s functional and anatomical data sets were processed, aligned and prepared in Brain Voyager (BV) for both within and between group random effects analyses based on steps described by Goebel et al. [46].

#### Preprocessing of functional data

Raw functional data (dicom format) was loaded and converted into Brain Voyager’s internal “FMR” data format. Standard sequence of preprocessing steps including slice scan time correction, head motion correction, drift removal and spatial smoothing with Gaussian filter (FWHM = 5 mm) were conducted for all paradigm data set.

#### Preprocessing of anatomical data

The anatomical data (dicom format) of each subject was loaded and converted into Brain Voyager’s internal “VMR” data format. Intensity inhomogeneities correction was applied and the data was then resampled to 1-mm resolution, and transformed into AC-PC and Talairach standard space. The three spatial transformations were combined and applied backward in one step to avoid quality loss due to successive data sampling. The two affine transformations, iso-voxel scaling and AC-PC transformation, were concatenated to form a single 4×4 transformation matrix. For each voxel coordinates in the target (Talairach) space a piece affine “Un-Talairah” step was performed, followed by application of the inverted spatial transformation matrix. The computed coordinates were used to sample the data points in the original 3-D space using sinc interpolation.

#### Brain segmentation

For 3-D visualization, the brain was segmented from surrounding head tissue using an automatic “brain peeling” tool. The tool analyzed the local intensity histogram in small volumes (20×20×20 voxels) to define thresholds for an adaptive region-growing technique. This step resulted in the automatic labeling of voxels containing the white and gray matter of the brain, but also other high-intensity head tissue. The next step consisted of a sequence of morphological erosions to remove tissue at the border of the segmented data. By “shrinking” the segmented data, this step separated subparts, which were connected by relatively thin “bridges” with each other. By determining the largest connected component after the erosion step, the brain was separated from other head tissue. Finally, the sequence of erosions was reversed but restricted to voxels in the neighborhood of the largest connected component.

#### Cortex segmentation

In order to perform a cortex-based data analysis, the gray/white matter boundary was segmented using largely automatic segmentation routines [47]. Following the correction of inhomogeneities in signal intensity across space as described above, the white/gray matter border was segmented with a region-growing method using an analysis of intensity histograms. Morphological operations were used to smooth the borders of the segmented data and to separate the left from the right hemisphere. Each segmented hemisphere was finally submitted to a “bridge removal” algorithm, ensuring the creation of topologically correct mesh representations [47]. The borders of the two resulting segmented subvolumes were tessellated to produce a surface reconstruction of the left and right hemispheres. For better visualization of the areas of activities including those in the sulcus, the resulting meshes were transformed into inflated cortical representations by performing repeated small morphing steps until the central sulcus were visible. The inflated cortical meshes were used as the reference meshes for functional data (maps and time courses) projection. For subsequent cortex-based analysis, the inflated cortical meshes were used to sample the functional data at each vertex (node), resulting in a mesh time course (“MTC”) dataset for each run of each subject.

#### Normalization of functional data

To transform the functional data into Talairach space, the functional time series data of each subject was first coregistered with the subject’s 3-D anatomical dataset, followed by the application of the same transformation steps as performed for the 3-D anatomical dataset (see above). This step results in normalized 4-D volume time course (“VTC”) data. In order to avoid quality loss due to successive data sampling, normalization was performed in a single step combining a functional-anatomical affine transformation matrix, a rigid-body AC-PC transformation matrix, and a piecewise affine Talairach grid scaling step. As described for the anatomical normalization procedure, these steps were performed backward, starting with a voxel in the Talairach space and sampling the corresponding data in the original functional space. In the context of the functional-anatomical alignment, some manual adjustment was necessary to reduce as much as possible the geometrical distortions of the echo-planar images, which exhibited linear scaling in the phase-encoding direction. The necessary scaling adjustment was done interactively using appropriate transformation and visualization tools of Brain Voyager QX.

#### Within- and between-group general linear model (GLM) analysis

Within-group random effect analysis was first conducted for each paradigm and regions of significant (P < 0.01 and cluster threshold > 150) activation (positively correlated BOLD) and deactivation (negatively correlated BOLD) were recorded. Between-group random effect analyses were also performed between three TG and the matched thermode presentations to assess the spatial summation effect of the grill on supraspinal thermal pain processing. In addition, between-group analyses were conducted to compare P46 and P40, HP and P46, and HP and P40 paradigms in HP related supraspinal regions. A protocol file (PRT) was derived representing the onset and duration of the events for the stimulation conditions. In order to account for hemodynamic delay and dispersion, each of the predictors was derived by convoluting an appropriate box-car waveform with a double-gamma hemodynamic response function [48] to extract brain regions with both positively and negatively correlated BOLD responses. False Discovery Rate (FDR) correction was used for multiple comparisons.

The spatial coordinates of significant clusters known as Volume of Interest (VOI) were first complied in a text file which was then subjected to anatomical naming via Talariach Client (http://www.talairach.org/client.html). The resulted anatomical regions were further verified with BV Tutor probabilistic anatomical map. For 3D cortical labeling, Patches of Interest (POI) were first selected using BVQX “POI Analysis Tool.” The POI’s were then converted to VOI’s to extrapolate the details of the defined region (including spatial coordinates) and classify them using the above mentioned VOI methodology.

#### Granger causality analysis

Granger Causality analysis (GCA) was conducted to explore the causal interaction (inference) among regions related to pain perception at each TG paradigm. The affected regions in the form of either activation or deactivation from the HP paradigm were used to create a cluster-based (anatomically based) template for the GCA in each paradigm. The template consisted of supraspinal regions related to acute HP. Each of the regions was used to estimate effective connectivity among clusters in each paradigm with the BV GCA plug-in. Using the average time course in one of the regions as a reference and the other regions as potential target regions of inference, computations were made to discern the correlation of the voxels in these regions from the rest of the brain. The result of the analysis was displayed as either positive values signifying significant influence directing from the reference cluster to the targeted regions or negative values representing the reverse direction [49]. In addition, clusters information including coordinates, sizes and Brodmann areas were converted by the Talairach Client into a text format after verifying the data with Brain Tutor [50,51]. The resulting text was imported to a spreadsheet and the network of inference was mapped onto a spatial representation of the brain network involved in acute thermal pain processing for each paradigm.

#### Psychological interactions analysis (PPI)

To further assess individual HP related supraspinal region’s functional connectivity to others, Psychophysiological Interaction(PPI) was conducted. First, on the Talairach group averaged “VMR”, the seed region of interest (ROI) for the PPI was loaded from the cluster-based (anatomically based) template from the HP paradigm. Each subject’s VTC data was loaded separately after which the ROI signal time course(RTC) was extracted as plain text. The design matrix (SDM: Single run Design Matrix), coding the exact timing of model predictors in the paradigm, was also extracted for each subject and saved as plain text. The SDM model predictors and RTC of the seed ROI were extracted and combined in an excel sheet where the average of each time course was calculated. New columns of data containing the result of the subtraction of the average value from the original value were considered demeaned. The PPI predictor was calculated by multiplying the demeaned model predictor with the demeaned RTC of the seed ROI. This PPI predictor data was used as a new predictor into the single study GLM dialog along with the original study predictors. To make sure that the scales of all predictors were represented exactly the same, all the predictors were z-transformed before running the GLM analysis. After running the GLM analysis, the interaction predictors were loaded as a contrast for each subject and each subject’s PPI volume map was saved. In the BrainVoyager Volume Maps dialog, all the saved maps for the group (n = 13) were loaded and averaged using the functions of the “Combine VMP” dialog. As the result, a new map was created in the main dialog representing the average interaction of the group. Clusters with P < 0.01 and cluster threshold > 100 voxels were selected. *Cluster information including coordinates, sizes and Brodmann areas were converted by the Talairach Client into a text format after verifying the data with Brain Tutor*[45,46,52,53]*.*

## Abbreviations

ACC: Anterior cingulate cortex; BA: Brodmann area; BOLD: Blood oxygen level dependent; BV: Brain voyager; DLPFC: Dorsolateral prefrontal cortex; FDR: False discovery rate; FMRI: Functional magnetic resonance imaging; FO: Frontal operculum; G2020: Thermal grill at 20°C; G2040: Thermal grill at 20° and 40°C; G4040: Thermal grill at 40°C; GCA: Granger causality analysis; GLM: General linear model; HP: Hot pain; IN: Insular cortex; IPL: Inferior parietal lobe; MPFC: Medial prefrontal cortex; P20: Thermal probe at 20°C; P40: Thermal probe at 40°C; P46: Thermal probe at 46°C; PFCs: Prefrontal cortices; POI: Patch of interest; SSC1: Primary somatosensory cortex; SSC2: Secondary somatosensory cortex; TG: Thermal grill; TH: Thalamus; VAS: Visual analogue scale; VOI: Volume of interest.

## Competing interests

The authors declare that they have no competing interests.

## Authors’ contributions

AL designed, conducted and performed data analysis of the study. AL also prepared the manuscript. SS conducted data analysis and assisted in manuscript preparation. EL assisted in conducting the study. JD assisted in conducting the study and initial data analysis. TY assisted in initial study design. All authors read and approved the final manuscript.

## References

[B1] Thunberg T Uppsala Lakforen Forh 1896 2 489

[B2] Craig AD Reiman EM Evans A Bushnell MC Functional imaging of an illusion of pain Nature 1996 384 258 260 10.1038/384258a0 8918874

[B3] Lindstedt F Lonsdorf TB Schalling M Kosek E Ingvar M Perception of thermal pain and the thermal grill illusion is associated with polymorphisms in the serotonin transporter gene PLoS One 2011 6 e17752 10.1371/journal.pone.0017752 21423614 PMC3057988

[B4] Craig AD Bushnell MC The thermal grill illusion: unmasking the burn of cold pain Science 1994 265 252 255 10.1126/science.8023144 8023144

[B5] Lindstedt F Johansson B Martinsen S Kosek E Fransson P Ingvar M Evidence for thalamic involvement in the thermal grill illusion: an FMRI study PLoS One 2011 6 e27075 10.1371/journal.pone.0027075 22096519 PMC3214046

[B6] Apkarian AV Bushnell MC Treede RD Zubieta JK Human brain mechanisms of pain perception and regulation in health and disease Eur J Pain 2005 9 463 484 10.1016/j.ejpain.2004.11.001 15979027

[B7] Tracey I Nociceptive processing in the human brain Curr Opin Neurobiol 2005 15 478 487 10.1016/j.conb.2005.06.010 16019203

[B8] Neugebauer V Galhardo V Maione S Mackey SC Forebrain pain mechanisms Brain Res Rev 2009 60 226 242 10.1016/j.brainresrev.2008.12.014 19162070 PMC2700838

[B9] Tracey I Neuroimaging of pain mechanisms Curr Opin Support Palliat Care 2007 1 109 116 10.1097/SPC.0b013e3282efc58b 18685351

[B10] Oshiro Y Quevedo AS McHaffie JG Kraft RA Coghill RC Brain mechanisms supporting spatial discrimination of pain J Neurosci 2007 27 3388 3394 10.1523/JNEUROSCI.5128-06.2007 17392455 PMC6672117

[B11] Seifert F Fuchs O Nickel FT Garcia M Dorfler A Schaller G Kornhuber J Sperling W Maihofner C A functional magnetic resonance imaging navigated repetitive transcranial magnetic stimulation study of the posterior parietal cortex in normal pain and hyperalgesia Neuroscience 2010 170 670 677 10.1016/j.neuroscience.2010.07.024 20643193

[B12] Moulton EA Pendse G Becerra LR Borsook D BOLD responses in somatosensory cortices better reflect heat sensation than pain J Neurosci 2012 32 6024 6031 10.1523/JNEUROSCI.0006-12.2012 22539862 PMC3347471

[B13] Bowsher D Leijon G Thuomas KA Central poststroke pain: correlation of MRI with clinical pain characteristics and sensory abnormalities Neurology 1998 51 1352 1358 10.1212/WNL.51.5.1352 9818859

[B14] Shukla S Torossian A Duann JR Leung A The analgesic effect of electroacupuncture on acute thermal pain perception–a central neural correlate study with fMRI Mol Pain 2011 7 45 10.1186/1744-8069-7-45 21645415 PMC3130679

[B15] Leung AY Wallace MS Schulteis G Yaksh TL Qualitative and quantitative characterization of the thermal grill Pain 2005 116 26 32 10.1016/j.pain.2005.03.026 15935558

[B16] Brooks JC Zambreanu L Godinez A Craig AD Tracey I Somatotopic organisation of the human insula to painful heat studied with high resolution functional imaging Neuroimage 2005 27 201 209 10.1016/j.neuroimage.2005.03.041 15921935

[B17] Bowsher D Representation of somatosensory modalities in pathways ascending from the spinal anterolateral funiculus to the thalamus demonstrated by lesions in man Eur Neurol 2005 54 14 22 10.1159/000086884 16015016

[B18] Brooks JC Nurmikko TJ Bimson WE Singh KD Roberts N fMRI of thermal pain: effects of stimulus laterality and attention Neuroimage 2002 15 293 301 10.1006/nimg.2001.0974 11798266

[B19] Tracey I Becerra L Chang I Breiter H Jenkins L Borsook D Gonzalez RG Noxious hot and cold stimulation produce common patterns of brain activation in humans: a functional magnetic resonance imaging study Neurosci Lett 2000 288 159 162 10.1016/S0304-3940(00)01224-6 10876085

[B20] Becerra L Iadarola M Borsook D CNS activation by noxious heat to the hand or foot: site-dependent delay in sensory but not emotion circuitry J Neurophysiol 2004 91 533 541 14715722 10.1152/jn.00326.2003

[B21] Neugebauer V Li W Processing of nociceptive mechanical and thermal information in central amygdala neurons with knee-joint input J Neurophysiol 2002 87 103 112 11784733 10.1152/jn.00264.2001

[B22] Neugebauer V Li W Differential sensitization of amygdala neurons to afferent inputs in a model of arthritic pain J Neurophysiol 2003 89 716 727 12574449 10.1152/jn.00799.2002

[B23] Tanimoto S Nakagawa T Yamauchi Y Minami M Satoh M Differential contributions of the basolateral and central nuclei of the amygdala in the negative affective component of chemical somatic and visceral pains in rats Eur J Neurosci 2003 18 2343 2350 10.1046/j.1460-9568.2003.02952.x 14622196

[B24] LeDoux J The emotional brain, fear, and the amygdala Cell Mol Neurobiol 2003 23 727 738 10.1023/A:1025048802629 14514027 PMC11530156

[B25] Bornhovd K Quante M Glauche V Bromm B Weiller C Buchel C Painful stimuli evoke different stimulus–response functions in the amygdala, prefrontal, insula and somatosensory cortex: a single-trial fMRI study Brain 2002 125 1326 1336 10.1093/brain/awf137 12023321

[B26] Brooks J Tracey I From nociception to pain perception: imaging the spinal and supraspinal pathways J Anat 2005 207 19 33 10.1111/j.1469-7580.2005.00428.x 16011543 PMC1571498

[B27] Youell PD Wise RG Bentley DE Dickinson MR King TA Tracey I Jones AK Lateralisation of nociceptive processing in the human brain: a functional magnetic resonance imaging study Neuroimage 2004 23 1068 1077 10.1016/j.neuroimage.2004.07.004 15528107

[B28] Graff-Guerrero A Gonzalez-Olvera J Fresan A Gomez-Martin D Mendez-Nunez JC Pellicer F Repetitive transcranial magnetic stimulation of dorsolateral prefrontal cortex increases tolerance to human experimental pain Brain Res Cogn Brain Res 2005 25 153 160 10.1016/j.cogbrainres.2005.05.002 15935625

[B29] Hardy SG Haigler HJ Prefrontal influences upon the midbrain: a possible route for pain modulation Brain Res 1985 339 285 293 10.1016/0006-8993(85)90094-0 4027627

[B30] Lorenz J Minoshima S Casey KL Keeping pain out of mind: the role of the dorsolateral prefrontal cortex in pain modulation Brain 2003 126 1079 1091 10.1093/brain/awg102 12690048

[B31] Wiech K Seymour B Kalisch R Stephan KE Koltzenburg M Driver J Dolan RJ Modulation of pain processing in hyperalgesia by cognitive demand Neuroimage 2005 27 59 69 10.1016/j.neuroimage.2005.03.044 15978845

[B32] Peltz E Seifert F DeCol R Dorfler A Schwab S Maihofner C Functional connectivity of the human insular cortex during noxious and innocuous thermal stimulation Neuroimage 2011 54 1324 1335 10.1016/j.neuroimage.2010.09.012 20851770

[B33] Tseng MT Tseng WY Chao CC Lin HE Hsieh ST Distinct and shared cerebral activations in processing innocuous versus noxious contact heat revealed by functional magnetic resonance imaging Hum Brain Mapp 2010 31 743 757 19823988 10.1002/hbm.20902 PMC6870753

[B34] Selvarajah D Wilkinson ID Gandhi R Griffiths PD Tesfaye S Microvascular perfusion abnormalities of the Thalamus in painful but not painless diabetic polyneuropathy: a clue to the pathogenesis of pain in type 1 diabetes Diabetes Care 2011 34 718 720 10.2337/dc10-1550 21282344 PMC3041213

[B35] Tseng MT Chiang MC Chao CC Tseng WY Hsieh ST fMRI evidence of degeneration-induced neuropathic pain in diabetes: enhanced limbic and striatal activations Hum Brain Mapp 2013 34 2733 2746 10.1002/hbm.22105 22522975 PMC6869882

[B36] Wager TD Atlas LY Lindquist MA Roy M Woo CW Kross E An fMRI-based neurologic signature of physical pain N Engl J Med 2013 368 1388 1397 10.1056/NEJMoa1204471 23574118 PMC3691100

[B37] Chen AC Niddam DM Crawford HJ Oostenveld R Arendt-Nielsen L Spatial summation of pain processing in the human brain as assessed by cerebral event related potentials Neurosci Lett 2002 328 190 194 10.1016/S0304-3940(02)00512-8 12133585

[B38] Peyron R Laurent B Garcia-Larrea L Functional imaging of brain responses to pain. A review and meta-analysis (2000) Neurophysiol Clin 2000 30 263 288 10.1016/S0987-7053(00)00227-6 11126640

[B39] Leung A Khadivi B Duann JR Cho ZH Yaksh T The effect of Ting point (tendinomuscular meridians) electroacupuncture on thermal pain: a model for studying the neuronal mechanism of acupuncture analgesia J Altern Complement Med 2005 11 653 661 10.1089/acm.2005.11.653 16131289

[B40] Leung A Li E Fallah A Schulteis G Novak E Duann JR Yaksh TL The effect of needle combination on the analgesic efficacy of the tendinomuscular meridians(TMM) systems Med Acupunct 2007 19 4 191 199 2007 10.1089/acu.2007.0564

[B41] Leung AY Kim SJ Schulteis G Yaksh T The effect of acupuncture duration on analgesia and peripheral sensory thresholds BMC Complement Altern Med 2008 8 18 10.1186/1472-6882-8-18 18452622 PMC2386116

[B42] Leung A Wallace MS Ridgeway B Yaksh T Concentration-effect relationship of intravenous alfentanil and ketamine on peripheral neurosensory thresholds, allodynia and hyperalgesia of neuropathic pain Pain 2001 91 177 187 10.1016/S0304-3959(00)00433-4 11240090

[B43] Becerra LR Breiter HC Stojanovic M Fishman S Edwards A Comite AR Gonzalez RG Borsook D Human brain activation under controlled thermal stimulation and habituation to noxious heat: an fMRI study Magn Reson Med 1999 41 1044 1057 10.1002/(SICI)1522-2594(199905)41:5<1044::AID-MRM25>3.0.CO;2-M 10332889

[B44] Ekblom A Hansson P Pain intensity measurements in patients with acute pain receiving afferent stimulation J Neurol Neurosurg Psychiatry 1988 51 481 486 10.1136/jnnp.51.4.481 3259976 PMC1032956

[B45] Leung A Duann JR Davis M Li E Fallah A Yaksh TL Facial-cervical collar restraint (FCCR) device in reducing head motion during a noxious stimulus study Neuroimage 2006 31 504

[B46] Goebel R Esposito F Formisano E Analysis of functional image analysis contest (FIAC) data with brainvoyager QX: From single-subject to cortically aligned group general linear model analysis and self-organizing group independent component analysis Hum Brain Mapp 2006 27 392 401 10.1002/hbm.20249 16596654 PMC6871277

[B47] Kriegeskorte N Goebel R An efficient algorithm for topologically correct segmentation of the cortical sheet in anatomical mr volumes Neuroimage 2001 14 329 346 10.1006/nimg.2001.0831 11467907

[B48] Friston KJ Fletcher P Josephs O Holmes A Rugg MD Turner R Event-related fMRI: characterizing differential responses Neuroimage 1998 7 30 40 10.1006/nimg.1997.0306 9500830

[B49] Abler B Roebroeck A Goebel R Hose A Schonfeldt-Lecuona C Hole G Walter H Investigating directed influences between activated brain areas in a motor-response task using fMRI Magn Reson Imaging 2006 24 181 185 10.1016/j.mri.2005.10.022 16455407

[B50] Lancaster JL Rainey LH Summerlin JL Freitas CS Fox PT Evans AC Toga AW Mazziotta JC Automated labeling of the human brain: a preliminary report on the development and evaluation of a forward-transform method Hum Brain Mapp 1997 5 238 242 10.1002/(SICI)1097-0193(1997)5:4<238::AID-HBM6>3.0.CO;2-4 20408222 PMC2860189

[B51] Lancaster JL Woldorff MG Parsons LM Liotti M Freitas CS Rainey L Kochunov PV Nickerson D Mikiten SA Fox PT Automated Talairach atlas labels for functional brain mapping Hum Brain Mapp 2000 10 120 131 10.1002/1097-0193(200007)10:3<120::AID-HBM30>3.0.CO;2-8 10912591 PMC6871915

[B52] O’Reilly JX Woolrich MW Behrens TE Smith SM Johansen-Berg H Tools of the trade: psychophysiological interactions and functional connectivity Soc Cogn Affect Neurosci 2012 7 604 609 10.1093/scan/nss055 22569188 PMC3375893

[B53] Yoshino A Okamoto Y Onoda K Yoshimura S Kunisato Y Demoto Y Okada G Yamawaki S Sadness enhances the experience of pain via neural activation in the anterior cingulate cortex and amygdala: an fMRI study Neuroimage 2010 50 1194 1201 10.1016/j.neuroimage.2009.11.079 19969094

